# Structural biology of the TRPM4 channel: architecture, modulation, and therapeutic opportunities

**DOI:** 10.3389/fphar.2026.1946877

**Published:** 2026-09-10

**Authors:** Ingrid Araya-Durán, Marena Rein, Daniella Delgado, Alejandro Yévenes, Victoria Flores del Pino, Fernando González-Nilo

**Affiliations:** 1 Center for Bioinformatics and Integrative Biology, Universidad Andrés Bello, Santiago, Chile; 2 Centro Interdisciplinario de Neurociencias de Valparaíso, Universidad de Valparaíso, Valparaíso, Chile

**Keywords:** Ca^2+^-activated cation channel, cryo-EM, gating, nanobodies, structural pharmacology, TRPM4

## Abstract

TRPM4 is a Ca^2+^-activated, monovalent-selective cation channel that conducts Na^+^ and K^+^ while remaining essentially impermeable to Ca^2+^. By translating cytosolic Ca^2+^ elevations into membrane depolarization rather than additional Ca^2+^ influx, TRPM4 operates as an electrical signal converter that tunes excitability, secretion, vascular tone, immune-cell behavior, and cell-death pathways. Dysregulated TRPM4 activity has been implicated in cardiac conduction disease, acute central nervous system injury and edema, and several cancers, making the channel a clinically relevant but mechanistically demanding target. Recent cryo-electron microscopy (cryo-EM) structures have transformed the field by resolving TRPM4 in closed, non-conductive intermediate, pre-open, open-pore, and drug-bound conformations, and by defining the allosteric interfaces that connect Ca^2+^ sensing, phosphoinositide binding, nucleotide inhibition, temperature-dependent remodeling, and pore opening. In parallel, pharmacology has advanced from low-selectivity tool compounds toward structurally localized inhibitors, direct agonists, extracellular antibodies, and prospective engineered binders whose mechanisms can be interpreted in state-dependent terms. Here, we synthesize this structural and pharmacological evidence to explain how TRPM4 architecture encodes modulation and gating, and how disease context should guide therapeutic strategy. We argue that the central challenge is no longer simply to activate or inhibit TRPM4, but to stabilize the right conformational state in the right tissue at the right stage of pathology. Finally, we discuss nanobodies as promising state-selective tools and potential biologics for next-generation TRPM4 modulation.

## 1 Introduction

Transient receptor potential (TRP) channels form a broad superfamily of cation channels that sense and integrate chemical, thermal, and mechanical cues across virtually every cell type. Within this superfamily, the melastatin (TRPM) subfamily is distinguished by large cytosolic domains and broad functional diversity, and TRPM4 is unusual even by TRPM standards. Encoded by the TRPM4 gene on chromosome 19 (Ciaglia et al., 2022), TRPM4 is a Ca^2+^-activated, monovalent-selective cation channel (Jimenez et al., 2020) that conducts Na^+^ and K^+^ but is essentially impermeable to Ca^2+^ (Liu et al., 2024). Together with its paralog TRPM5, TRPM4 defines a distinctive subgroup of TRP channels that are activated by intracellular Ca^2+^ yet selectively permeable to monovalent cations, with a reported unitary conductance of approximately 25 pS (Liedtke and Heller, 2007). TRPM4 activation requires an increase in intracellular Ca^2+^, with reported EC_50_ values of approximately 0.5–3 µM (Barbet et al., 2008). Once activated, Na^+^ influx depolarizes the membrane and reduces the electrochemical driving force for sustained Ca^2+^ entry, allowing TRPM4 to function as a negative-feedback regulator of Ca^2+^ signaling (Souza Bomfim et al., 2022). Thus, because TRPM4 changes the electrical state of the membrane without providing a route for Ca^2+^ entry, it functions less as a classical ion-influx pathway and more as a signal converter that reshapes excitability, secretion, migration, osmotic-stress responses, and cell-death programs across many tissues (Abriel et al., 2012; Dienes et al., 2021).

TRPM4 is broadly expressed in many tissues, including the heart, prostate, colon and intestine, pancreas, kidney, and skeletal muscle (Kruse et al., 2009; Berg et al., 2016; Dwyer et al., 2011; Cheng et al., 2007; Flannery et al., 2015; Brinkmeier, 2011). It is detected at lower levels in immune-related tissues such as the thymus and spleen (Fonfria et al., 2006). Functional studies have further implicated TRPM4 in immune cells, including T cells, dendritic cells, and mast cells, as well as in vascular smooth muscle, where it contributes to the regulation of cerebral arterial tone (Barbet et al., 2008; Earley et al., 2004; Launay et al., 2004; Vennekens et al., 2007). These diverse physiological settings are relevant because changes in expression, trafficking, gating, or regulatory environment can reshape TRPM4 function. Inherited TRPM4 variants have been linked to cardiac conduction disorders and arrhythmias (Kruse et al., 2009; Liu et al., 2013; Vandewiele et al., 2022); in CNS injury, upregulated TRPM4, often discussed in association with SUR1, has been associated with edema and secondary tissue damage (Jha et al., 2021; Mehta et al., 2015; Stokum et al., 2023); and altered TRPM4 abundance has been reported in several malignancies, including prostate, colorectal, and breast cancers, diffuse large B-cell lymphoma, and acute myeloid leukemia, among others (Berg et al., 2016; Guo et al., 2025; Kappel et al., 2019; Loo et al., 2017; Wang et al., 2020).

Over the past decade, three converging lines of research have reshaped how TRPM4 is understood and pursued as a therapeutic target. First, the structural landscape has expanded dramatically: cryo-EM studies of human and other mammalian TRPM4 channels provide an increasingly detailed conformational itinerary for this Ca^2+^-activated channel (Autzen et al., 2018; Duan et al., 2018; Ekundayo et al., 2025; Guo et al., 2017; Hu et al., 2024; Teixeira-Duarte et al., 2026a; Teixeira-Duarte et al., 2026b; Winkler et al., 2017). Second, building on these structural advances, TRPM4 pharmacology has evolved from low-selectivity tool compounds toward more targeted inhibitors, direct agonists, and extracellular antibodies whose mechanisms of action can increasingly be linked to defined binding sites (Ekundayo et al., 2025; Grand et al., 2008; Liu et al., 2026; Ozhathil et al., 2018; Teixeira-Duarte et al., 2026a; Wei et al., 2022; 2025). Third, evidence from human genetics, cancer biology, and injury models has established TRPM4 as more than a channel of mechanistic interest: it is a clinically relevant membrane protein whose functional output depends on the structural integration of Ca^2+^, lipids, nucleotides, temperature, interacting proteins, and disease-associated sequence variation (Kappel et al., 2019; Stokum et al., 2023).

Consequently, recent structural and pharmacological advances have provided a clearer framework for understanding the molecular mechanisms that control TRPM4 gating and modulation. Here, we review how endogenous regulators, conformational transitions, and ligand interactions shape TRPM4 function, and how this knowledge may guide the development of more selective therapeutic strategies. We also discuss remaining challenges, including the need to define TRPM4 regulation in specific cellular contexts and disease states. Finally, we consider how antibodies, particularly nanobodies, may offer new opportunities for precise, state-dependent modulation of TRPM4 activity.

## 2 Structural architecture of TRPM4

The expanding repertoire of TRPM4 structures deposited in the Protein Data Bank has substantially refined our understanding of its overall architecture, gating, and ligand-dependent regulation (Winkler et al., 2017; Guo et al., 2017; Autzen et al., 2018; Duan et al., 2018). Table 1 summarizes the progression from the initial cryo-EM structures of TRPM4 to more recent structures that capture distinct steps in its activation and modulation pathways (Winkler et al., 2017; Guo et al., 2017; Hu et al., 2024; Teixeira-Duarte et al., 2026b; Hu et al., 2026). To avoid overlap between structural and functional terminology, pore conformation and ligand occupancy are considered separately here. Within this framework, the structural landscape of TRPM4 comprises apo-closed conformations, Ca^2+^-bound closed or non-conductive intermediates, and open-pore conformations, whereas Ca^2+^, PtdIns(4,5)P_2_ (PIP_2_), ATP, and pharmacological modulators are specified independently as bound ligands. A Ca^2+^-bound non-conductive conformation has been interpreted as a putative desensitized state, whereas related Ca^2+^-bound closed structures have been characterized as pre-open intermediates, indicating that “desensitized” is best regarded as a functional assignment rather than a mutually exclusive structural category (Hu et al., 2024; Teixeira-Duarte et al., 2026b). Open TRPM4 conformations can be stabilized by Ca^2+^ together with either PIP_2_ or the positive modulator decavanadate (DVT), whereas ATP stabilizes a closed conformation and the anthranilic-acid inhibitors NBA and CBA can trap a Ca^2+^-primed, non-conductive pre-open state at physiological temperature (Hu et al., 2024; Teixeira-Duarte et al., 2026b; Hu et al., 2026). Recent studies further show that temperature reshapes TRPM4 conformation and ligand recognition, emphasizing that ligand occupancy alone does not uniquely define the conductive state of the channel (Hu et al., 2024; Hu et al., 2026). Thus, these structures provide a structural foundation for interpreting TRPM4 gating, state-dependent pharmacology, and emerging therapeutic opportunities.

**TABLE 1:** Cryo-EM structures of TRPM4. Representative TRPM4 structures deposited in the Protein Data Bank are organized by structural pore conformation separately from ligand/regulatory occupancy and experimental context. This separation avoids treating ligand-bound, desensitized, inhibited, or inactivated as mutually exclusive conformational states. Functional terms are retained only in the structural/functional interpretation column when explicitly supported by the original study.

PDB	References	Species	Res. (Å)	Pore conformation	Ligand/regulatory occupancy	Main finding
30KH	(Schneiter et al., 2026)	*Homo sapiens*	3.70	Closed	No added modulator; native membrane vesicles; 8 °C; endogenous cholesterol/lipids	Preserved native-like cholesterol/paralipid environment
30KW			3.10	Closed	No added modulator; GDN; 8 °C	GDN-solubilized apo reference with conserved cholesterol-binding pattern
30KZ			3.40	Closed	PBA; 8 °C	PBA binds the canonical anthranilic-inhibitor pocket.
30 LA			3.00	Closed	Ca^2+^; 37 °C	Defined Ca^2+^/temperature effects on the regulatory region
30LD			3.00	Closed	Ca^2+^ + PBA; 37 °C	PBA relocates near the Ca^2+^ site at physiological temperature
5WP6	(Winkler et al., 2017)	*Homo sapiens*	3.80	Closed	Ca^2+^ + DVT; detergent-solubilized	Defined human TRPM4 architecture and two DVT-binding sites
6BCJ	(Guo et al., 2017)	*Mus musculus*	3.14	Closed	No ATP; Na^+^ observed	First mouse TRPM4 structure defining three-tiered architecture
6BCL			3.54	Closed	No ATP; extended coiled-coil model	Resolved extended coiled coil supporting tetramer assembly
6BCO			2.88	Closed	ATP-bound	Identified the inhibitory ATP site and local allosteric changes
6BCQ			3.25	Closed	ATP-bound; extended coiled-coil model	Confirmed ATP-inhibited architecture with extended coiled coil
6BQV	(Autzen et al., 2018)	*Homo sapiens*	3.10	Closed	Ca^2+^-bound; lipid nanodisc	Identified the primary Ca^2+^ site that primes opening
6BQR			3.20	Closed	Ca^2+^-free; lipid nanodisc	Ca^2+^-free nanodisc reference for activation comparisons
6BWI	(Duan et al., 2018)	*Homo sapiens*	3.70	Closed	Na^+^ and lipid/CHS-associated	Mapped Na^+^ sites, gates, glycosylation, disulfide, and lipids
8RCR	(Ekundayo et al., 2025)	*Homo sapiens*	3.60	Closed	No inhibitor; native SMA lipid nanodisc	Native-lipid apo reference revealing cholesterol near inhibitor pocket.
8RCU			3.50	Closed	IBA-bound; native SMA lipid nanodisc	Validated the IBA-binding pocket as an inhibitor site
8RD9			4.30	Closed	NBA-bound; native SMA lipid nanodisc	Located NBA in the transmembrane inhibitory pocket.
9B8W	(Hu et al., 2024)	*Homo sapiens*	3.10	Closed; warm/pre-open-like	Ca^2+^; 37 °C	Identified the warm conformation and intracellular Ca^2+^ site
9B92			3.50	Closed; cold	Ca^2+^; low-temperature condition	Low-temperature Ca^2+^-bound reference for the cold state
9B8X			3.00	Closed (parent state)	Ca^2+^; 37 °C; focused/subunit reconstruction	Refined ICD rearrangements during Ca^2+^-dependent warming
9B8Y			3.20	Open pore	Ca^2+^ + DVT; 37 °C	Captured DVT-stabilized opening at physiological temperature
9B8Z			3.40	Open pore (parent state)	Ca^2+^ + DVT; 37 °C; focused/subunit reconstruction	Refined the warm DVT site coupled to pore opening
9B90			3.40	Closed	Ca^2+^ + ATP; 37 °C	Defined warm-specific ATP binding and pore closure
9B91			3.30	Closed (parent state)	Ca^2+^ + ATP; 37 °C; focused/subunit reconstruction	Refined temperature-dependent ATP recognition
9B93			3.10	Closed; cold	Ca^2+^-free/EDTA	Ca^2+^ is required for the warm conformational transition
9B94			3.10	Closed; cold	Ca^2+^; E396 A mutant	E396 is required for Ca^2+^-dependent warm-state formation
9MRT	(Teixeira-Duarte et al., 2026b)	*Homo sapiens*	2.44	Open pore	Ca^2+^ + PIP_2_	Defined Ca^2+^–PIP_2_-dependent opening and PIP_2_ binding
9MT8			3.19	Closed/non-conductive	Ca^2+^-bound	Captured a Ca^2+^-bound desensitized closed-pore state
9MTA			2.78	Closed	Apo	High-resolution apo closed reference structure
9MTC			2.63	Closed	ATP-bound	Defined ATP inhibition and allosteric pore closure
9I3R	(Pugh et al., 2025)	*Homo sapiens*	3.46	Closed	Endogenous Ca^2+^ + cholesterol/lipids; MAASTY native nanodisc	MAASTY nanodiscs preserved endogenous lipids and Ca^2+^ binding
9SMK			3.80	Closed	Ca^2+^ + CHS; MSP2N2 nanodisc	Synthetic nanodisc comparator with similar closed-pore architecture
9Y2B	(Liu et al., 2026)	*Homo sapiens*	3.20	Closed; pre-open intermediate	DAB (Ca^2+^-free); physiological temperature	Identified DAB as a Ca^2+^-independent agonist
9Y2A			3.30	Closed; pre-open intermediate	DAB + Ca^2+^; physiological temperature	DAB activates TRPM4 through a TMD-centered pathway
9Y2C			3.40	Closed (parent state)	DAB + Ca^2+^; focused/subunit reconstruction	Refined the DAB pocket and local TMD rearrangements
9YVK	(Teixeira-Duarte et al., 2026a)	*Homo sapiens*	2.69	Closed; pre-open intermediate	NC1; Ca^2+^-free condition	NC1 induces Ca^2+^-like rearrangements but requires PIP_2_ for opening
9YVL			2.78	Open pore	NC1 + PIP_2_	PIP_2_ couples NC1-induced movements to pore opening
9YVM			2.73	Open pore	NC1 + PIP_2_ + EGTA	NC1 and PIP_2_ open TRPM4 independently of Ca^2+^
9YVN		*Mus musculus*	3.42	Non-conductive/closed-like	NC1	NC1 drives a nonconductive mouse TRPM4 state
9YVO			2.91	Non-conductive/closed-like	NC1 + PIP_2_	PIP_2_ fails to rescue NC1-inactivated mouse TRPM4
9YVP			2.81	Open pore	NC1 + PIP_2_; humanizing triple mutant	Humanizing mutations restore NC1-sensitive opening in mouse TRPM4
9Z1W	(Hu et al., 2026)	*Homo sapiens*	2.86	Closed; agonist-bound/pre-open-like	Ca^2+^ + TPPO; 37 °C	TPPO acts as a Ca^2+^/temperature-dependent agonist
9Z1X			3.00	Closed (parent state)	Ca^2+^ + TPPO; 37 °C; warm TMD-focused reconstruction	Defined TPPO binding and Ca^2+^ synergy at 37 °C
9Z1Y			3.26	Closed (parent state)	Ca^2+^ + TPPO; 37 °C; cold TMD-focused reconstruction	Captured coexisting cold and warm TPPO-bound conformations
9Z1Z			2.63	Closed; cold	Ca^2+^ + TPPO; 18 °C	Low temperature limits productive TPPO agonism
9Z20			2.51	Closed; cold	TPPO + EGTA; 37 °C	TPPO requires Ca^2+^ to stabilize the warm agonist state
9Z21			2.83	Closed; agonist-bound/pre-open-like	Ca^2+^ + NC1; 37 °C	High Ca^2+^ remodels NC1 binding and limits activation
9Z22			2.58	Closed; agonist-bound/pre-open-like	NC1 + EGTA; 37 °C	Captured Ca^2+^-independent NC1 binding in the upper pocket.
9Z23			2.59	Closed; non-conductive pre-open	Ca^2+^ + CBA; 37 °C	CBA locks TRPM4 in a nonconductive pre-open state
9Z24			3.10	Closed (parent state)	Ca^2+^ + CBA; 37 °C; TMD-focused reconstruction	Refined the CBA lower-pocket binding site
9Z27			2.76	Closed; non-conductive pre-open	Ca^2+^ + CBA + DVT; 37 °C	CBA blocks DVT binding and prevents pore opening
9Z25			2.64	Closed; non-conductive pre-open	Ca^2+^ + NBA; 37 °C	NBA stabilizes a nonconductive pre-open inhibited state
9Z26			2.90	Closed (parent state)	Ca^2+^ + NBA; 37 °C; TMD-focused reconstruction	Defined the NBA site relative to the upper agonist pocket.

“Closed,” “open pore,” and “closed/non-conductive pre-open” describe structural pore conformations or structure-based intermediates. “Desensitized,” “inhibited,” and “inactivated” are functional assignments and are therefore not used as stand-alone pore-state categories.

Abbreviations: Å, ångström; ATP, adenosine triphosphate; CBA, 4-chloro-2-(2-(2-chlorophenoxy)acetamido)benzoic acid; CHS, cholesteryl hemisuccinate; DAB, deacetyl bisacodyl; DVT, decavanadate; EDTA, ethylenediaminetetraacetic acid; EGTA, ethylene glycol-bis(β-aminoethyl ether)-N,N,N′,N′-tetraacetic acid; GDN, glyco-diosgenin; IBA, 4-chloro-2-(2-(3-iodophenoxy)acetamido)benzoic acid; ICD, intracellular domain; MAASTY, poly (methacrylic acid-co-styrene); MSP2N2, membrane scaffold protein 2N2; NBA, 4-chloro-2-(2-(naphthalene-1-yloxy)acetamido)benzoic acid; NC1, Necrocide 1; PBA, 4-chloro-2-(2-(3-(prop-2-yn-1-yloxy)phenoxy)acetamido)benzoic acid; PIP_2_, phosphatidylinositol 4,5-bisphosphate; Res, resolution; SMA, styrene–maleic acid; TMD, transmembrane domain; TPPO, triphenylphosphine oxide.

**FIGURE 1:**
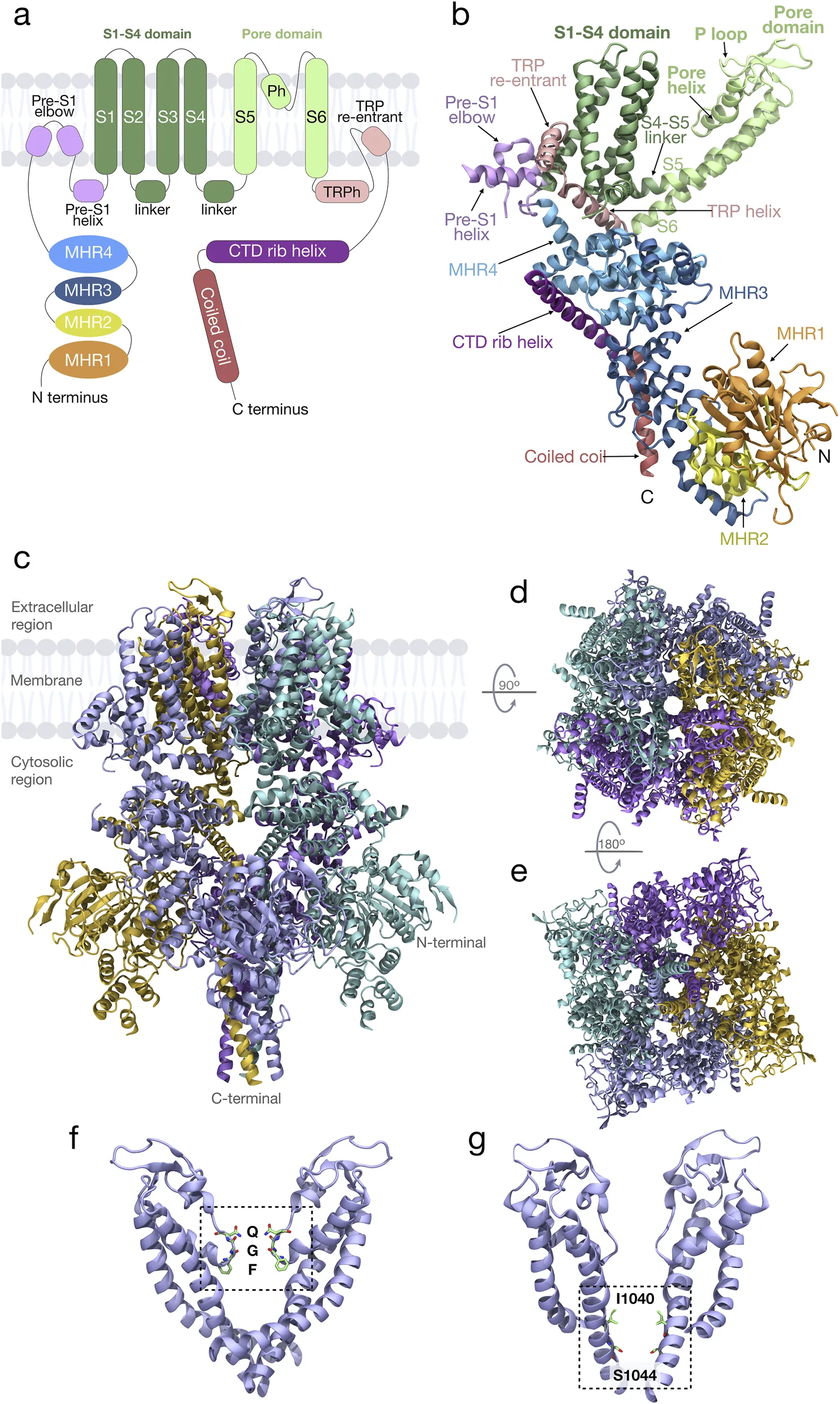
Structural architecture and pore organization of TRPM4. **(a)** Schematic topology of a single TRPM4 subunit, showing the cytosolic N-terminal MHR1–MHR4 domains, the pre-S1 helix and elbow, the six transmembrane helices organized into the S1–S4 sensor-like domain and the S5–S6 pore domain, the pore helix and pore loop, and the C-terminal TRP helix, TRP re-entrant segment, CTD rib helix, and coiled-coil region. **(b)** Three-dimensional representation of a TRPM4 protomer highlighting the same structural elements in the folded channel architecture. **(c)** Lateral view of the TRPM4 homotetramer embedded in the membrane, with the extracellular region oriented toward the top and the cytosolic region toward the bottom. Gray bars indicate the approximate boundaries of the lipid bilayer. The channel is organized into three major structural layers: the membrane-embedded transmembrane domain, the large cytosolic N-terminal scaffold, and the C-terminal assembly. Each protomer is shown in a distinct color. **(d)** Extracellular view of the tetramer, highlighting the arrangement of the transmembrane domains around the central ion-conduction pathway. **(e)** Cytosolic view showing the crown-like organization of the intracellular MHR scaffold and the central C-terminal coiled-coil assembly that contributes to tetramer stabilization. **(f)** Zoomed view of the upper, extracellular-facing pore region, showing the FGQ-associated constriction formed by residues F975, G976, and Q977 in human TRPM4. **(g)** Zoomed view of the lower intracellular gate, highlighting the cytosolic constriction formed by I1040 and S1044 in human TRPM4. Panels a–e were generated from PDB ID 6BCQ, whereas panels f and g were generated from PDB ID 9 MRT. All molecular images were generated using VMD 1.9.3 (Humphrey et al., 1996).

TRPM4 is a homotetrameric ion channel of approximately 500 kDa (Guo et al., 2017; Winkler et al., 2017). At the protomer level, TRPM4 adopts the three-layered organization characteristic of the TRPM family: a large cytosolic N-terminal domain, a membrane-embedded transmembrane domain (TMD), and a C-terminal domain (CTD) comprising the TRP helix, rib-like connectors, and a distal coiled coil, as shown in Figures 1a, b (Duan et al., 2018; Guo et al., 2017; Winkler et al., 2017). Within each subunit, the N-terminal cytosolic region contains four TRPM-homology regions (MHR1–MHR4), the membrane region contains six transmembrane helices (S1–S6), and the C-terminal region contributes the TRP helix, rib helices, and coiled coil (Figure 1c) (Duan et al., 2018; Teixeira-Duarte et al., 2026b; Winkler et al., 2017). The following sections describe these structural layers in detail, providing the framework for understanding how endogenous ligands regulate TRPM4 conformation, gating, and modulation.

### 2.1 Cytosolic domain

#### 2.1.1 N-terminal domain

The N-terminal domain forms a large cytosolic scaffold that surrounds and interacts with C-terminal elements in the tetramer and is organized into the four MHRs characteristic of the TRPM subfamily (Teixeira-Duarte et al., 2026b; Winkler et al., 2017). MHR1 and MHR2 sit furthest from the membrane and form a single compact, bi-lobed subdomain (MHR1/2) built from an eight-stranded β-sheet core surrounded by eight α-helices (Winkler et al., 2017). This region is also referred to as the nucleotide-binding domain (NBD) for its ability to bind ATP; within it, a small ankyrin-like repeat domain (ARD) contributes two ankyrin-like repeats that form a rigid unit with the NBD (Autzen et al., 2018; Guo et al., 2017).

MHR3 and MHR4 are composed of stacked α-helices and sit closer to the membrane, with MHR3 bridging MHR1/2 and MHR4, and MHR4 connecting to the TMD (Autzen et al., 2018; Winkler et al., 2017). MHR4 is considered a signal-transducing module because it “clasps” the TRP domain and engages the S2–S3 linker and rib helix of the CTD, coupling cytosolic conformational changes to the gating machinery (Winkler et al., 2017). Between neighboring protomers, MHR1 of one subunit interacts with MHR3 of the adjacent subunit to form a wide aperture that houses, but does not directly contact, the C-terminal coiled-coil helices (Guo et al., 2017).

#### 2.1.2 C-terminal domain

The CTD provides the structural foundation for tetrameric assembly and a critical surface for allosteric modulation. It is often described as an “umbrella-like” architecture positioned directly beneath the ion-conducting pore. In human structures, the proximal C-terminus contains the TRP helix, which connects to S6 and runs parallel to the inner membrane; more distally, elongated rib-like helices and a central coiled-coil stabilize the tetramer (Duan et al., 2018; Winkler et al., 2017).

Functionally, the TRP helix is a key feature of the CTD, serving as a critical coupling element that links conformational changes in the sensor domains to opening of the S6 gate. Structural transitions in the TMD converge onto the TRP helix, which then propagates conformational change into the cytosolic scaffold and back into the pore (Duan et al., 2018; Teixeira-Duarte et al., 2026b; Winkler et al., 2017). The TRP helix is divided into two segments: TRP helix one runs parallel to the cytosolic membrane surface and contains the highly conserved “TRP box” motif. In contrast, TRP helix 2 (the TRP re-entrant segment) comprises a short loop embedded between S1 and S2 and a returning helix on the cytoplasmic side. From the TRP domain, a rib (“stretcher”) helix extends horizontally toward the symmetry axis in each protomer; in the tetramer, these four helices form the “ribs” of the umbrella, threading through tunnels formed by the N-terminal MHRs and tethering them into a crown-like assembly (Guo et al., 2017; Winkler et al., 2017). Connected to the rib helices, a coiled-coil “pole” forms the vertical shaft of the umbrella: four parallel α-helices wind into a tetrameric bundle that penetrates the central hole of the N-terminal assembly and is essential for assembly specificity (Duan et al., 2018; Guo et al., 2017; Winkler et al., 2017).

### 2.2 Transmembrane domain

The TMD represents a major pharmacological target because ligand binding within transmembrane pockets can directly modulate pore opening and ion conduction. Its six membrane-spanning helices (S1–S6) are organized into two functionally distinct modules: the S1–S4 voltage-sensing-like domain (VSD) and a neighboring pore domain built from S5, the pore helix and loop, and S6 (Autzen et al., 2018; Duan et al., 2018; Guo et al., 2017). As in other domain-swapped TRP channels, the S1–S4 bundle of one subunit communicates with the pore domain of the adjacent subunit through the S4–S5 linker and the TRP helix, creating the principal pathway by which ligand-dependent conformational changes reach the gate (Duan et al., 2018; Teixeira-Duarte et al., 2026b).

Importantly, however, domain swapping in TRP channels may not necessarily represent a fixed architectural feature. Recent cryo-EM and hydrogen–deuterium exchange mass-spectrometry analyses of TRPM8 in cell-derived membrane vesicles identified a dynamic equilibrium between the conventional fully swapped architecture and a distinct semi-swapped configuration, in which repositioning of S6 and the outer pore alters the intersubunit organization without requiring tetramer disassembly (Choi et al., 2026). Both configurations were observed in avian and human TRPM8, and menthol shifted the conformational ensemble toward the semi-swapped population; moreover, the study linked these rearrangements to activation-related changes in the pore and to the energetic basis of cold sensitivity. These observations support the concept that alternative domain-swapping configurations can form part of a physiologically relevant gating landscape rather than representing only static differences between channel structures. For TRPM4, however, no equivalent semi-swapped or non-swapped architecture has yet been resolved. Available structures obtained in detergent, reconstituted lipid nanodiscs, endogenous-lipid nanodiscs, and across multiple ligand- and gating-associated conditions have retained the canonical domain-swapped arrangement (Autzen et al., 2018; Hu et al., 2024; Ekundayo et al., 2025; Pugh et al., 2025; Teixeira-Duarte et al., 2026b). Thus, whether TRPM4 transiently samples alternative intersubunit arrangements in native membranes or tissue-specific environments remains an open question that will require direct structural and functional investigation.

#### 2.2.1 The S1–S4 sensor-like module

The S1–S4 region is best described as a sensor-like module rather than a classical voltage sensor. Although it adopts the overall fold of a VSD, TRPM4 shows only weak intrinsic voltage dependence because S4 lacks the regularly spaced positive charges of canonical voltage-gated channels, containing a single conserved basic residue (Arg905 in human TRPM4) (Duan et al., 2018; Guo et al., 2017; Winkler et al., 2017). Structural work therefore favors the view that this region functions less as an autonomous voltage sensor than as a membrane-embedded signaling hub whose behavior is shaped by Ca^2+^, lipids, and channel state. A distinctive feature is the pre-S1 “elbow” and “shoulder,” membrane-proximal elements that wrap around the S1–S4 bundle at the inner leaflet and belong to the transmembrane coupling apparatus, where they are well placed to participate in lipid-sensitive allosteric control. The canonical transmembrane Ca^2+^ site sits at the intracellular face of this same region, reinforcing the picture of the S1–S4 module as a ligand-responsive control surface rather than a pure voltage sensor (Autzen et al., 2018; Teixeira-Duarte et al., 2026b).

#### 2.2.2 Pore architecture and ion selectivity

The TRPM4 pore is formed by the S5–S6 region of the four subunits, including the re-entrant pore loop and pore helix, and contains two principal constriction sites (Figures 1d, e). The upper constriction lies around the conserved FGQ motif (notably F975, G976, and Q977) (Figure 1f), whereas the lower, intracellular gate is formed by I1040 and S1044 (Figure 1g) in the human channel (Autzen et al., 2018; Duan et al., 2018; Winkler et al., 2017). In the apo structures, this lower S6 gate is too narrow to permit ion permeation, whereas the selectivity filter is comparatively wide, consistent with the unusual pore architecture of a channel that conducts hydrated monovalent cations while excluding divalent ions (Guo et al., 2017; Duan et al., 2018). Earlier mutagenesis further established the selectivity-filter glutamine as a major determinant of TRPM4 monovalent selectivity, because substitution of the corresponding Q977 residue alters the monovalent permeability sequence and can introduce measurable Ca^2+^ permeability (Nilius et al., 2005; Ullrich et al., 2005).

More recent open-state structures have clarified which pore rearrangements accompany TRPM4 activation. In Ca^2+^-bound closed or pre-open conformations, substantial rearrangements can occur in the cytosolic domains and S1–S4 coupling machinery while the pore itself remains closed. By contrast, opening stabilized either by Ca^2+^ and DVT at physiological temperature or by Ca^2+^ and PIP_2_ involves outward displacement of the pore-lining S6 helices and dilation of the intracellular gate. In the Ca^2+^/DVT-bound warm structure, for example, the pore reaches a radius of approximately 2.5 Å, whereas the more recent Ca^2+^/PIP_2_ structure shows that movement of the S4–S5/S5 coupling region drives S6 away from the pore axis, translating I1040 and S1044 outward to generate a conductive gate (Hu et al., 2024; Teixeira-Duarte et al., 2026b). Thus, TRPM4 opening is associated primarily with dilation of the lower S6 gate rather than with a major rearrangement of the selectivity-filter region.

Comparison with TRPM5 is most informative at this pore-mechanistic level. TRPM5 contains the homologous FGQ (904-906) selectivity-filter motif and an intracellular gate centered on I966. Cryo-EM structures of zebrafish TRPM5 captured both apo-closed and Ca^2+^-bound open conformations, showing that activation expands the narrowest radius at the I966 lower gate from approximately 0.8 Å to 2.7 Å, while the extracellular pore loop and selectivity-filter region undergo comparatively little rearrangement (Ruan et al., 2021). Molecular-dynamics simulations further indicate that TRPM5 monovalent selectivity does not arise simply from steric exclusion at the FGQ filter. Instead, an uncharged and relatively hydrophobic transition zone between the selectivity filter and central cavity—termed a “hydrophobic funnel”—creates a larger energetic barrier for divalent Ca^2+^ than for Na^+^ or K^+^. Hydrophobic residues immediately below the filter, including F904 and I903, together with hydrophobic S6 residues lining the cavity, contribute to this barrier (Ives et al., 2024). Importantly, the corresponding filter and transition-zone sequence is highly conserved in TRPM4, and Na^+^ densities have been resolved within the central cavity of several TRPM4 structures, suggesting that a related permeation mechanism may operate in both monovalent-selective channels; however, this hydrophobic-funnel mechanism has so far been demonstrated directly by simulation for TRPM5 rather than TRPM4 (Ives et al., 2024).

### 2.3 Endogenous modulators and binding surfaces

**FIGURE 2:**
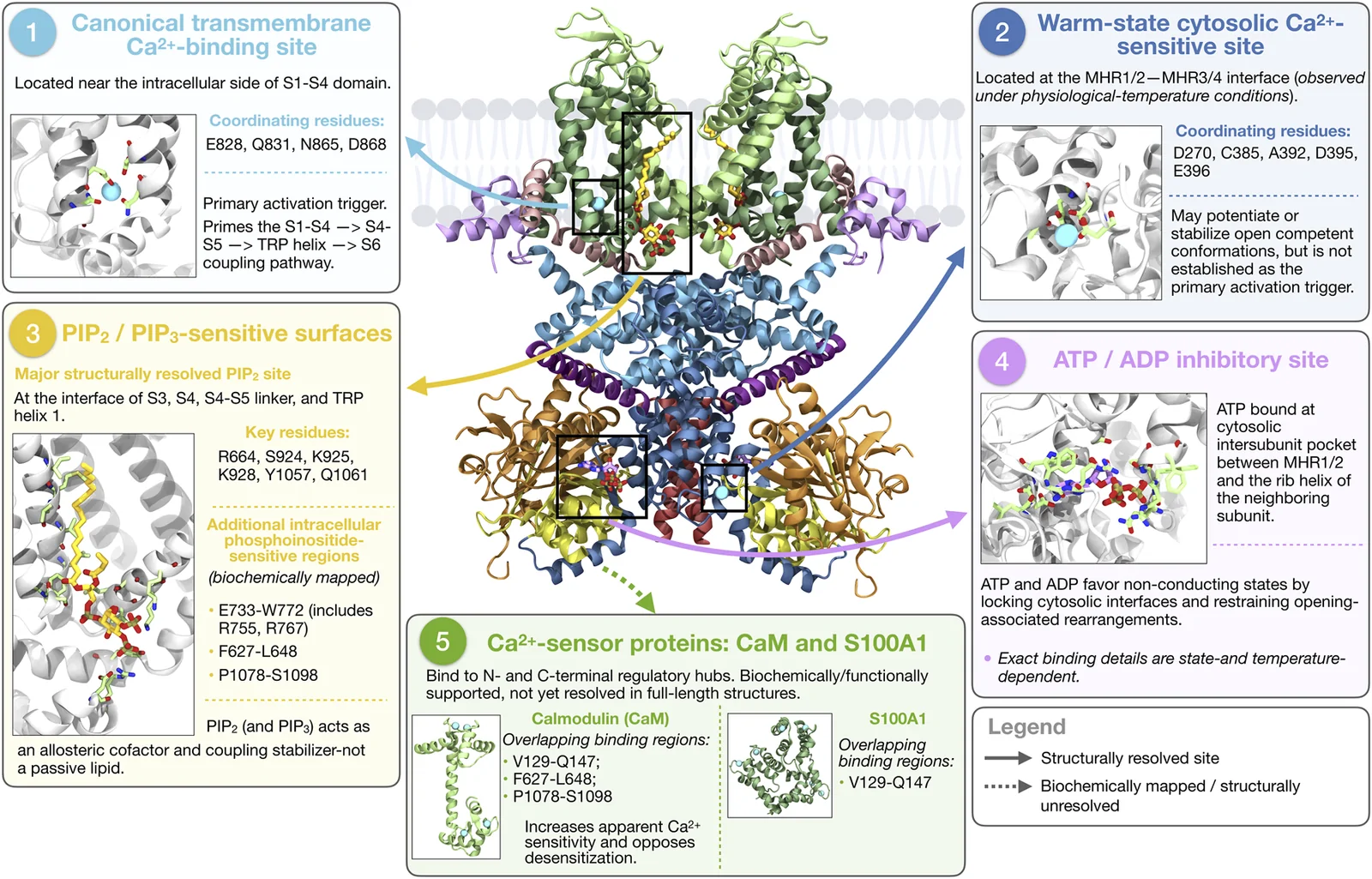
Endogenous modulatory surfaces of TRPM4. Schematic overview of the principal endogenous regulatory surfaces that shape TRPM4 gating. The figure highlights the canonical transmembrane Ca^2+^-binding site, a warm-state cytosolic Ca^2+^-sensitive site, phosphoinositide-sensitive regions, the ATP/ADP inhibitory interface, and cytosolic regulatory hubs for CaM and S100A1. These sites illustrate how TRPM4 integrates ions, lipids, nucleotides, and Ca^2+^-sensor proteins through distributed allosteric surfaces. Solid annotations indicate structurally resolved sites, whereas dashed annotations denote biochemically mapped or structurally unresolved interactions. TRPM4 and ligand positions were derived using PDB IDs 9B8W, 9 MRT, and 9MTC; calmodulin and S100A1 were represented using PDB IDs 3CLN and 5K89, respectively, and images were generated using VMD 1.9.3 (Humphrey et al., 1996).

TRPM4 gating is controlled by a distributed network of endogenous modulators that bind to both transmembrane and cytosolic regulatory surfaces (Figure 2). This section summarizes how Ca^2+^, phosphoinositides, nucleotides, and Ca^2+^-sensor proteins shape channel state, coupling efficiency, and desensitization.

#### 2.3.1 Calcium

The canonical Ca^2+^-binding site is positioned within the transmembrane sensor-like domain, near the intracellular side of S1–S4 (Figure 2, panel 1). Structures of human TRPM4 in lipid nanodiscs identified a pocket coordinated mainly by E828, Q831, N865, and D868 (Autzen et al., 2018). This site offers a clear structural explanation for the established observation that TRPM4 is activated by increases in cytosolic Ca^2+^: ion coordination at this pocket reorganizes the local sensor-like region and primes the downstream coupling pathway that extends through S4, the S4–S5 linker, and the TRP helix toward the S6 gate (Autzen et al., 2018; Teixeira-Duarte et al., 2026b). Physiological temperature studies expanded this picture: at 37 °C, TRPM4 adopts a “warm” conformation that reveals a second, intracellular Ca^2+^-sensitive site within the cytosolic domain rather than the transmembrane core (Figure 2, panel 2). This warm-state site forms at the interface between the MHR1/2 and MHR3/4 domains. During the cold-to-warm transition, a rigid-body movement of MHR1/2 toward MHR3/4 brings helix α12 of MHR1/2 into proximity with helix α13 of MHR3/4, thereby creating the Ca^2+^-coordinating pocket involving D270, C385, A392, D395, and E396 (Hu et al., 2024). Together, these observations suggest that the canonical transmembrane site is the primary activation trigger, whereas the warm-state cytosolic site helps potentiate or stabilize open-competent conformations; current data do not indicate that the second site alone drives TRPM4 into a pre-open state in the way the canonical site does (Hu et al., 2024; Teixeira-Duarte et al., 2026b).

#### 2.3.2 Phospholipids (PIP_2_/PIP_3_)

Electrophysiological studies first revealed the strong dependence of TRPM4 activity on phosphoinositides, and recent structures have explained how lipid binding supports channel activation. In excised patches, TRPM4 desensitizes rapidly after membrane excision. However, exogenous PIP_2_ rescues run-down currents, restores activity, and markedly increases apparent Ca^2+^ sensitivity—arguing that PIP_2_ is not a minor permissive lipid but as an allosteric cofactor that stabilizes Ca^2+^-responsive channel conformations (Nilius et al., 2006; Zhang et al., 2005). Biochemical studies then showed that TRPM4 carries multiple intracellular phosphoinositide-sensitive surfaces rather than a single discrete motif. A proximal N-terminal segment (E733–W772) binds both PIP_2_ and PIP_3_, with R755 and R767 prominent in recognition (Bousova et al., 2015), and later mapping identified additional epitopes in both intracellular termini—particularly F627–L648 in the N-terminus and P1078–S1098 in the C-terminus, the latter showing especially strong PIP_2_ affinity (Bousova et al., 2020) (Figure 2, panel 3).

Recent structural work supplied the missing mechanistic link by localizing a major phospholipid-binding site to the transmembrane–cytosolic coupling region near S3, S4, the S4–S5 linker, and the TRP helix, where residues including R664, S924, K925, K928, Y1057, and Q1061 coordinate the lipid and stabilize the coupling geometry required for a conductive open state (Figure 2, panel 3). In the absence of this interaction, Ca^2+^ can still occupy its transmembrane site. However, the channel readily collapses into a desensitized, non-conducting conformation, highlighting the role of PIP_2_ as a key stabilizer of the activated state rather than a passive membrane component (Teixeira-Duarte et al., 2026b). Taken together with biochemical mapping of phosphoinositide-sensitive regions and with recent evidence linking heat- and PIP_2_-dependent regulation to human disease (Bousova et al., 2020; Tian et al., 2026), PIP_2_ is best regarded as a core allosteric cofactor of TRPM4 gating.

#### 2.3.3 Nucleotides (ATP/ADP)

Nucleotide regulation of TRPM4 is more complex than a simple inhibitory mechanism. Early functional work established that free ATP and ADP inhibit the channel. In contrast, Mg-ATP helps restore activity and Ca^2+^ sensitivity after desensitization, indicating that the effect depends strongly on chemical state and channel context. ADP was the most potent adenine nucleotide tested, inhibiting TRPM4 in the low-micromolar range (Nilius et al., 2005; Nilius et al., 2004b). The first structural explanation came from mouse TRPM4 showing that ATP binds to an intersubunit cytosolic pocket within the N-terminal region, where the adenine moiety was coordinated in part by aromatic residues, including H160, W214, and F228, and ATP binding was proposed to restrain the cytosolic rearrangements required for opening (Guo et al., 2017). Physiological-temperature human structures later revised this picture, identifying a distinct warm-state ATP site shifted by ∼20 Å from the earlier low-temperature assignment and positioned between the MHR1/2 region and the rib helix of the neighboring subunit (Hu et al., 2024) (Figure 2, panel 4). The most up-to-date multi-state human structures show ATP stabilizing non-conducting conformations by locking cytosolic interfaces and preventing the changes required for pore activation (Teixeira-Duarte et al., 2026b). ATP is therefore best understood as a state-dependent negative regulator whose binding details must be interpreted in the context of temperature and channel state.

#### 2.3.4 Ca^2+^-sensor proteins (calmodulin and S100A1)

Ca^2+^-sensor proteins detect changes in intracellular calcium through EF-hand motifs and, upon binding, undergo conformational changes that allow them to regulate target proteins such as TRPM4, allowing cells to translate Ca^2+^ signals into specific responses (Figure 2, panel 5). Although no full-length cryo-EM structure of TRPM4 in complex with a Ca^2+^-sensor protein is yet available, biochemical and functional studies have defined key features of the interaction. Calmodulin (CaM) increases apparent Ca^2+^ sensitivity, suppresses desensitization, and is required for normal activation, placing it as an important stabilizer of the responsive state; five Ca^2+^-dependent CaM-binding sites were originally identified in the cytosolic domain (two N-terminal, three C-terminal), with functional disruption pointing most strongly to the C-terminal sites (Nilius et al., 2005). Later work mapped three principal CaM-binding regions: an N-terminal site (V129–Q147), a second N-terminal site (F627–L648), and a C-terminal site (P1078–S1098) (Bousova et al., 2020; Vydra Bousova et al., 2023). The first contains a 1-5-10 hydrophobic CaM-recognition motif conserved between TRPM4 and TRPM5, suggesting a shared regulatory mechanism; reported affinities are in the low-micromolar range, consistent with specific but reversible engagement rather than a permanently bound structural component (Vydra Bousova et al., 2023).

In addition to CaM, S100A1 has emerged as a second endogenous Ca^2+^-sensor implicated in TRPM4 regulation. It binds the distal N-terminal segment V129–Q147, overlapping the CaM site, with hydrophobic residues L134, L138, and V143 and basic residues R139, R140, and R144 contributing to binding; the reported affinity is again low-micromolar, supporting a role as a genuine modulator rather than an incidental interactor (Bousova et al., 2018). Crucially, residue-level mapping reveals that the same terminal surfaces overlap not only between CaM and S100A1 but also with PIP_2_-binding epitopes (including F627–L648 and P1078–S1098), implying that the TRPM4 N- and C-termini act as multifunctional regulatory hubs where several ligands compete to set channel state (Bousova et al., 2018; 2020; Vydra Bousova et al., 2023) (Figure 2, panels 3 and 5). As discussed below, the presence of multiple regulatory interfaces on accessible cytosolic surfaces makes TRPM4 an attractive target for engineered binders.

#### 2.3.5 Polyamines

Endogenous polyamines represent an additional cytosolic regulatory input, but their molecular binding site remains unresolved. Early electrophysiological work showed that intracellular polyamines inhibit TRPM4b (the long splice variant of the TRPM4 channel), together with intracellular nucleotides, indicating that positively charged metabolites can negatively regulate Ca^2+^-activated TRPM4 currents (Nilius et al., 2004b). Given their polycationic nature, polyamines may act through electrostatic interactions with cytosolic regulatory regions, acidic intracellular surfaces, or pore-proximal elements; this remains to be tested structurally.

## 3 Gating mechanisms

Current evidence supports a stepwise state-dependent gating cycle in which Ca^2+^ acts as the initial trigger, PIP_2_ serves as the principal open-state stabilizer, CaM acts as a sensitizing, anti-desensitization factor, and ATP favors non-conducting conformations (Autzen et al., 2018; Hu et al., 2024; Nilius et al., 2005; 2006; Teixeira-Duarte et al., 2026b; Zhang et al., 2005). The key conceptual point is that Ca^2+^ binding and pore opening are not the same event. Ca^2+^ first promotes the channel into an activated, open-competent configuration, but stable conduction additionally requires that the coupling pathway from the S1–S4 module through the S4–S5 linker and TRP helix to S6 remain intact (Autzen et al., 2018; Teixeira-Duarte et al., 2026b).

It begins at the canonical transmembrane Ca^2+^ pocket formed by E828, Q831, N865, and D868. Ca^2+^ binding shifts the S1–S4 module into an activated arrangement in which the N-terminal part of S3 swings inward, W864 is displaced, and the motion is transmitted through S4 toward the neighboring pore module. This is best described as a primed, open-competent state rather than a fully open one: the lower S6 gate can remain shut, so Ca^2+^ occupancy alone does not guarantee conduction (Autzen et al., 2018; Teixeira-Duarte et al., 2026b). Indeed, the human Ca^2+^-only particle resolved in the recent multi-state study is more accurately interpreted as a desensitized-after-activation state than as a simple pre-open intermediate: in this conformation, the crucial F910–F935 hydrophobic contact that normally couples sensor movement to pore opening becomes disengaged, so the conformational changes triggered by Ca^2+^ binding are no longer efficiently transmitted to the S6 gate (Teixeira-Duarte et al., 2026b). Thus, the channel remains Ca^2+^-activated but fails to sustain the conformational coupling required to stabilize a conductive open state.

PIP_2_ is the ligand that most effectively stabilizes the transition from this activated-but-fragile condition to a true open state. Early electrophysiology showed that TRPM4 currents run down rapidly after patch excision even in the presence of Ca^2+^, and that exogenous PIP_2_—or Mg-ATP-driven phosphoinositide resynthesis—can rescue activity; single-channel analysis showed that run-down reflects a collapse in open probability rather than a change in conductance, with openings becoming brief and infrequent until PIP_2_ restores long bursts (Zhang et al., 2005). This rescue is now structurally explained by the PIP_2_ site at the junction of S3, S4, the S4–S5 linker, and TRP helix 1, where the lipid acts as a molecular bridge that stabilizes the productive coupling geometry and prevents the F910–F935 slip associated with desensitization. In this sense, the true open state of TRPM4 is best described as a “Ca^2+^-plus-PIP_2_” state in which the lower S6 gate is held outward and the pore remains conductive (Teixeira-Duarte et al., 2026b). The same logic explains why PIP_2_ shifts TRPM4 activity into a more physiological voltage range: it does not replace Ca^2+^ as the activating ligand but makes Ca^2+^-driven opening far easier to achieve and maintain, increasing apparent Ca^2+^ sensitivity by roughly two orders of magnitude and shifting voltage dependence strongly toward more negative potentials, so that current no longer depends on strong depolarization (Nilius et al., 2006).

CaM appears to support the same mechanism, although the evidence is functional rather than structurally resolved. CaM binding at C-terminal sites increases apparent Ca^2+^ sensitivity, shifts activation toward less depolarized voltages. It slows desensitization in inside-out patches—functionally similar to PIP_2_ in opposing desensitization and preserving an open-competent state. In the absence of a resolved structure, however, CaM is safer regarded as a sensitizing cofactor than as a structurally defined open-state clamp equivalent to PIP_2_ (Nilius et al., 2005).

ATP contributes a distinct, inhibitory arm of the cycle. Recent human structures place ATP at a cytosolic intersubunit interface, where it acts as a molecular “glue” that fastens MHR elements together and prevents the upward, rotational movement of the intracellular domain associated with opening. Physiological-temperature work reached a parallel conclusion, with ATP trapping a non-conducting warm conformation rather than simply displacing Ca^2+^. In the state-resolved analysis, ATP could lock the channel in a closed conformation even in the presence of Ca^2+^ and PIP_2_, indicating that it strongly favors and stabilizes non-conducting states and can override productive, Ca^2+^/PIP_2_-supported opening (Hu et al., 2024; Teixeira-Duarte et al., 2026b). Whether ATP can bind a fully open channel and then close it—or instead acts only on activated-but-not-yet-open intermediates—remains unresolved. The practical conclusion is that ATP, like temperature, must be treated as part of the state landscape rather than as a simple on/off switch.

## 4 Structural pharmacology of TRPM4

**FIGURE 3:**
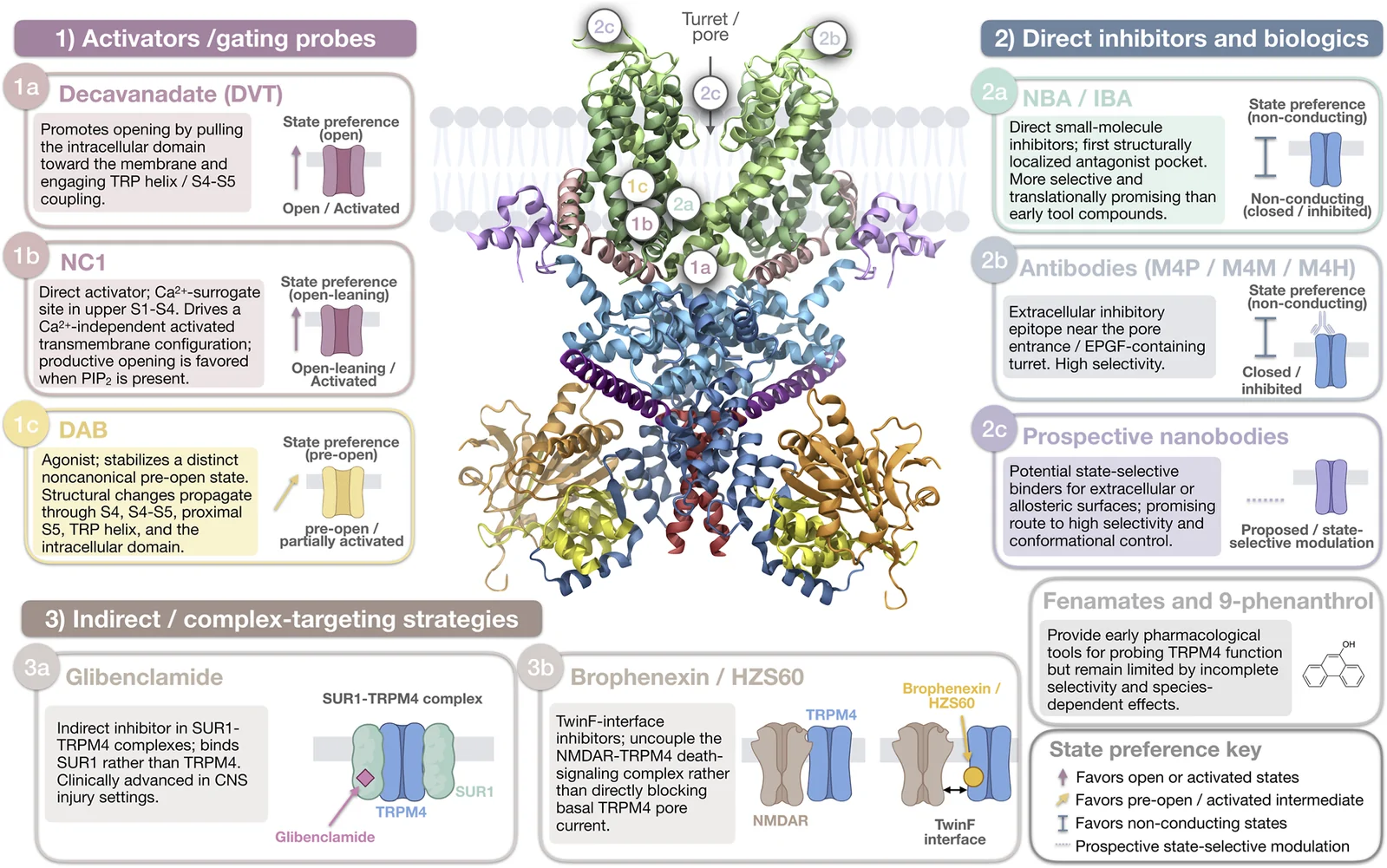
Representative modulators and sites of action on TRPM4. Schematic overview of the structural pharmacology of TRPM4, highlighting known and proposed sites of action for representative activators, inhibitors, biologics, and indirect modulatory strategies. Historical tool compounds, such as fenamates and 9-phenanthrol, provided early pharmacological tools for probing TRPM4 function. State-preference icons summarize whether each modulator favors open/activated, pre-open/intermediate, non-conducting, or prospective state-selective conformations. Images were generated using VMD 1.9.3 (Humphrey et al., 1996), and the icons were created with BioRender.com.

The expanding structural framework of TRPM4 has transformed its pharmacology from a collection of broad tool compounds into a more mechanism-informed landscape of activators, inhibitors, biologics, and indirect modulatory strategies. Recent cryo-EM structures have shown that modulation of TRPM4 is influenced by multiple regulatory surfaces rather than just one druggable site. These surfaces are spread across the transmembrane domain, the TRP helix, the S4–S5 linker, the intracellular MHR regions, and the extracellular vestibule. These sites are functionally coupled to channel state, lipid environment, Ca^2+^ occupancy, temperature, and conformational transitions between closed, pre-open, desensitized, inhibited, and open states. As a result, pharmacological modulation of TRPM4 must be interpreted in a structural context: compounds that appear similar functionally may act through distinct pockets, whereas ligands occupying adjacent or overlapping sites may stabilize different gating outcomes depending on the conformational ensemble available under a given experimental or physiological condition. Figure 3 provides an overview of the known and proposed sites at which TRPM4 modulators act within the channel architecture. It highlights their preferred states, providing a structural map that clarifies how small molecules, antibodies, and indirect regulatory methods can be used to achieve more selective and therapeutically beneficial control of TRPM4 activity.

### 4.1 Activators

One of the earliest characterized activators of TRPM4 was decavanadate (DVT) (Figure 3, panel 1a). Early electrophysiological studies showed that DVT acts from the intracellular side and strongly suppresses voltage-dependent deactivation, promoting opening at negative potentials (Nilius et al., 2004a). More recent structural work localized the functionally relevant warm-state DVT site to a cavity at the transmembrane–intracellular interface, where the polyanion is coordinated by several basic residues (R597, R601, K613, R664, K925, K928, and K1049); binding pulls the intracellular domain toward the membrane and transmits conformational changes through the TRP helix and S4–S5 coupling machinery to drive outward S6 movement and pore opening (Figure 3, panel 1a). Despite its mechanistic value, DVT is limited as a drug scaffold: it is a large, highly anionic intracellular ligand unlikely to cross membranes efficiently, and its polyanionic character may promote nonspecific electrostatic interactions—consistent with the additional, apparently nonspecific cold-state DVT sites observed structurally (Hu et al., 2024). DVT is therefore best viewed as a probe of TRPM4 gating, although the functionally relevant pocket it revealed may still inform drug design.

A more drug-like and mechanistically precise activator emerged with Necrocide 1 (NC1) (Figure 3, panel 1b). Whereas DVT mainly served as a gating probe, NC1 is a direct small-molecule activator and thus a more informative starting point for structural pharmacology (Fu et al., 2025). Structural work placed NC1 in the upper S1–S4 region above the canonical Ca^2+^ site, where it acts as a non-competitive surrogate for Ca^2+^ and drives a Ca^2+^-independent activated transmembrane configuration that can be converted into a fully open state when PIP_2_ is present (Figure 3, panel 1b). NC1 activity is strongly conditioned by intrinsic Ca^2+^: activation is favored at low resting Ca^2+^, whereas higher Ca^2+^ progressively disfavors productive binding through allosteric remodeling of the pocket (Hu et al., 2026). Functionally, NC1 drives prolonged Na^+^ entry, osmotic ballooning, and necrotic cell death—the Na^+^ Entry–driven Cell death by Necrosis Sodium Overload (NECSO) phenotype (Teixeira-Duarte et al., 2026a). This finding presents both an opportunity and a cautionary note. In cancer cells with high TRPM4 expression, such activity might be therapeutically exploitable, but in normal tissue, it defines a clear safety boundary for agonist design.

Deacetyl-bisacodyl (DAB), like NC1, binds within the S1–S4 transmembrane gating hub above the canonical Ca^2+^ site, but in a neighboring, partially overlapping pocket (Liu et al., 2026; Teixeira-Duarte et al., 2026a) (Figure 3, panel 1c). DAB also promotes a Ca^2+^-independent pre-open state, but, unlike NC1, it does not simply reproduce the canonical Ca^2+^-triggered intermediate; instead, it stabilizes a distinct, noncanonical partially activated conformation in which structural changes propagate through S4, the S4–S5 linker, proximal S5, the TRP helix, and the intracellular domain, yet still do not fully open the pore in the resolved structure (Figure 3, panel 1c). Nevertheless, DAB is clearly a functional agonist: it evokes robust, persistent Ca^2+^-independent TRPM4 currents, raises intracellular Na^+^, and drives TRPM4-dependent intestinal fluid secretion *in vivo*, effects that are in global and intestinal-epithelial TRPM4 knockouts (Liu et al., 2026). Although no DAB-plus-PIP_2_ open-state structure has yet been reported, the functional data strongly suggest that DAB can support a stable conductive state in native membranes, likely aided by endogenous lipids absent from the structural preparations.

### 4.2 Direct small-molecule inhibitors

The development of direct TRPM4 inhibitors has progressed from broad-acting compounds to more selective, channel-targeted molecules (Figure 3, panel 2a and lower-right inset). Flufenamic acid and related fenamates, such as meclofenamate, were important in the pre-structure era because they provided the field its first pharmacological leverage on TRPM4-like currents in native systems (Dienes et al., 2021; Guinamard et al., 2013; Kovács et al., 2022). Flufenamic acid was especially useful because TRPM4 shows relatively high sensitivity to this compound, with low-micromolar inhibition in heterologous and native preparations. However, these compounds never solved the selectivity problem: fenamates act broadly on other TRP channels and on many non-TRP conductance, so they could support a case for TRPM4 involvement but not provide clean target attribution (Guinamard et al., 2013; Preti et al., 2022).

9-Phenanthrol marked the next step as the first widely adopted, a more TRPM4-focused pharmacological tool (Figure 3, lower-right inset). In the original heterologous assays, 9-phenanthrol reversibly blocked human TRPM4 with micromolar potency while sparing TRPM5 (Grand et al., 2008), thereby helping establish roles for TRPM4 in atrial electrophysiology, and vascular tone (Hof et al., 2013; Simard et al., 2013). Later studies, however, revealed important species-dependent effects and limitations in selectivity. At concentrations commonly used to inhibit TRPM4, 9-phenanthrol can also inhibit the TMEM16 A chloride channel, making it difficult to assign all observed physiological effects specifically to TRPM4 (Arullampalam et al., 2021; Chen Y. et al., 2019; Dienes et al., 2021; Low et al., 2021). This compound is useful for studying TRPM4, but it should not be considered a good starting point for drug development.

A major advance came from the anthranilic-acid series (Figure 3, panel 2a). Ozhathil and colleagues identified halogenated anthranilic amides with markedly improved potency and selectivity relative to flufenamic acid and 9-phenanthrol, including low-micromolar and submicromolar compounds that moved the field from channel probing toward genuine medicinal chemistry (Ozhathil et al., 2018; Preti et al., 2022). That transition gained force once the inhibitory mechanism could be localized structurally: Ekundayo and colleagues placed NBA and IBA in a membrane-proximal pocket near S3, S4, the S4–S5 linker, and the TRP helix, providing the first direct structural foothold for human TRPM4 antagonist design (Figure 3, panel 2a) (Ekundayo et al., 2025). Species dependence nonetheless remains a major translational caution. CBA and 9-phenanthrol do not behave equivalently on human and mouse TRPM4, and intracellular 9-phenanthrol can even potentiate mouse TRPM4 under some conditions, whereas NBA is far more consistent across orthologs. These findings indicate that direct antagonism of TRPM4 has finally become structurally interpretable and chemically tractable, but meaningful translation will require state-aware pharmacology and explicit attention to species differences rather than uncritical extrapolation from mouse data (Arullampalam et al., 2021; Ekundayo et al., 2025; Hu et al., 2026).

### 4.3 Antibody-based inhibitors

Antibody-mediated inhibition differs from small-molecule antagonism in two important ways: it does not require access to a buried transmembrane pocket, instead targeting accessible extracellular regions, and prolonged antibody exposure can reduce surface TRPM4 abundance in addition to acute functional inhibition (Figure 3, panel 2b) (Chen B. et al., 2019; Low et al., 2021; Wei et al., 2025). The first reported example was the rodent polyclonal antibody M4P, which targeted an extracellular, pore-proximal epitope, reduced TRPM4 current, and was protective in stroke models (Chen B. et al., 2019; Wei et al., 2020). The field subsequently shifted toward human monoclonal antibodies as a more translational approach to TRPM4 targeting. Low and colleagues developed M4M against a short extracellular human TRPM4 sequence near the pore entrance and showed that it suppresses TRPM4 currents and reduces swelling in human brain-microvascular endothelial cells (Low et al., 2021); subsequent epitope mapping localized the core recognition motif to the extracellular, EPGF-containing turret region, establishing that TRPM4 presents a structurally accessible external inhibitory surface (Figure 3, panel 2b) (Wei et al., 2022). Humanization of the M4M lineage yielded M4H, which retained nanomolar peptide-binding affinity and blocked human TRPM4 currents *in vitro* with low microgram-per-milliliter potency (Wei et al., 2025). From a structural pharmacology standpoint, the main advantage of this class is the high selectivity conferred by an exposed extracellular epitope; however, the best-characterized epitopes are species-biased, complicating conventional rodent validation and making translation highly dependent on model choice (Low et al., 2021; Wei et al., 2022; 2025).

### 4.4 Indirect inhibitors

A third class of TRPM4-targeting strategies reduces TRPM4-dependent signaling without occupying a defined TRPM4 antagonist pocket, by either suppressing TRPM4-containing complexes through a partner subunit or disrupting pathogenic signaling assemblies that require TRPM4 (Figure 3, panels 3a and 3b). Glibenclamide exemplifies the first mechanism: in the SUR1–TRPM4 complex, it binds the regulatory subunit SUR1 rather than TRPM4 itself, and co-assembly with SUR1 confers a glibenclamide sensitivity that TRPM4 alone largely lacks (Figure 3, panel 3a) (Jha et al., 2021; Woo et al., 2013; Pergakis et al., 2019). It is therefore an indirect inhibitor of TRPM4-dependent current, especially in CNS injury, where SUR1–TRPM4 is induced, and represents the most clinically advanced TRPM4-linked modulator to date, having entered randomized trials in CNS injury (Jha et al., 2021; Sheth et al., 2024). The second mechanism is represented by TwinF-interface inhibitors, Brophenexin and HZS60, designed to uncouple the NMDAR–TRPM4 death-signaling complex rather than to block basal TRPM4 pore current (Figure 3, panel 3b) (Casby et al., 2024; 2026; Sun et al., 2025; Yan and Bading, 2023). HZS60 improved brain penetration and ischemia-model performance relative to earlier compounds yet did not measurably inhibit baseline Ca^2+^-activated TRPM4 currents in the reported assays (Sun et al., 2025), underscoring that “TRPM4-directed” therapy need does not necessarily require direct pore block.

## 5 Outlook

### 5.1 Nanobodies as state-selective modulators of TRPM4

The pharmacology surveyed above converges on a single, demanding conclusion: the most valuable future TRPM4 therapeutics will not be generic blockers but agents that recognize a particular conformational state and a particular structural surface. Small molecules struggle in both aspects. The functionally relevant pockets for Ca^2+^, PIP_2_, ATP, and the synthetic agonists are largely buried at transmembrane–cytosolic interfaces; selectivity against TRPM5 and unrelated channels is hard-won; and ortholog-specific behavior repeatedly undermines rodent-to-human translation (Arullampalam et al., 2021; Ekundayo et al., 2025; Hu et al., 2026). Biologics may overcome some of these limitations by targeting TRPM4 through distinct mechanisms. Earlier antibody studies suggest that TRPM4 has extracellular epitopes accessible for selective targeting; however, the best-characterized site appears to be specific to humans (Low et al., 2021; Wei et al., 2022; 2025). Accordingly, a logical next step is to move from full-length antibodies toward smaller, more versatile engineered binders, with nanobodies representing a particularly attractive option.

Nanobodies (VHH single-domain antibodies, ∼12–15 kDa) are compact antigen-binding domains derived from camelid heavy-chain-only antibodies (Hamers-Casterman et al., 1993). Related single-domain antibody scaffolds, including shark-derived VNAR domains (Greenberg et al., 1995), share this small modular architecture and expand the repertoire of engineered binders for targeting challenging protein surfaces. Their small size, high thermodynamic stability, and simple single-domain organization confer several advantages over conventional IgG antibodies for ion-channel targeting: long and often protruding antigen-binding loops capable of accessing recessed or cryptic epitopes; superior tissue and tumor penetration; straightforward recombinant production; and exceptional engineerability into multivalent, bispecific, or fusion formats (Debie et al., 2020; Jovčevska and Muyldermans, 2020; Kang et al., 2021). Critically, nanobodies frequently recognize specific conformational states, which makes them not only candidate therapeutics but also powerful conformational stabilizers for structural studies of gating intermediates (Ali et al., 2025). For an allosterically complex, state-cycling channel such as TRPM4, the ability to lock or report on a defined conformation is exactly the property the field has identified as most valuable (Huffer et al., 2024).

Although TRPM4-specific nanobody structures have not yet been reported, several ion-channel structures in complex with nanobodies or nanobody-derived binders have demonstrated the feasibility of using these small scaffolds to stabilize or modulate specific channel conformations (Table 2). Examples include 5-HT3 receptors (Hassaine et al., 2014), GABAA receptors (Laverty et al., 2019), pentameric ligand-gated ion channel ELIC (Brams et al., 2020; Hénault et al., 2019), α7 nicotinic receptors (Prevost et al., 2023), ASIC1a (Wu et al., 2021), Kv1.3 (Selvakumar et al., 2022), TREK-2 (Rödström et al., 2024), LRRC8/VRAC (Deneka et al., 2021) and TMEM175 channels (Brunner et al., 2020). In pentameric ligand-gated channels, nanobodies have been engineered to act as both positive and negative allosteric modulators, binding a vestibule site, partly shared with small-molecule modulators, and extrapolating to the human 5-HT_3_ receptor (Brams et al., 2020). For the T-cell channel Kv1.3, a nanobody suppresses activity by binding to the voltage-sensing and pore domains, thereby stabilizing an inactive conformation (Selvakumar et al., 2022). For the α7 nicotinic receptor, two closely related nanobodies function as a positive allosteric modulator and a silent allosteric ligand, which control the global allosteric state of the receptor through a shared apical epitope (Prevost et al., 2023). Moreover, for the volume-regulated LRRC8 channels, a panel of synthetic nanobodies (“sybodies”) either inhibits or enhances activity depending on which epitope of the cytoplasmic domain they engage (Deneka et al., 2021). Collectively, these studies illustrate that nanobodies can achieve bidirectional, epitope-defined, and state-biased modulation by engaging allosteric surfaces distant from the permeation pathway, thereby offering the precise conformational control that TRPM4 may require (Figure 3, panel 2c).

**TABLE 2:** Representative PDB structures of ion channels in complex with nanobodies or related engineered binders. These examples illustrate the use of nanobodies, megabodies, sybodies, and nanobody-fusion constructs as conformational stabilizers, allosteric modulators, crystallization chaperones, and structural tools across diverse ion-channel families.

PDB	Ion channel	Binder	Comment
4PIR	Mouse serotonin 5-HT3 receptor	Nanobody VHH15 (*Lama glama*)	VHH15 caps the extracellular domain near the orthosteric serotonin-binding site, helping stabilize the receptor for crystallographic analysis and providing one of the first high-resolution structural views of a mammalian Cys-loop receptor
6I53	Human GABA_A α1β3γ2 receptor	Megabody38 (*Lama glama*)	This nanobody-derived megabody acts as a positive allosteric modulator and conformational stabilizer, helping resolve the full-length receptor in a desensitized state and illustrating the utility of engineered binders for structural studies
6SSI/6SSP/6HJX/6HJY	Pentameric ligand-gated ion channel ELIC	Nanobodies PAM/NAM/Nb72 (*Lama glama*)	The PAM and NAM nanobodies illustrate how extracellular binders can positively or negatively tune pLGIC gating through allosteric sites rather than by directly occluding the pore, whereas Nb72 highlights the use of nanobodies as crystallization/stabilization tools to resolve ELIC conformations and lipid-associated structural features
8CE4/8CI1/8C9X/8CAU/8CI2	Human α7 nicotinic acetylcholine receptor	Nanobodies E3/C4 (*Vicugna pacos*)	Both nanobodies bind a common extracellular epitope at the apex of the receptor, involving adjacent subunits. E3 acts as a positive allosteric modulator that stabilizes an agonist-bound-like extracellular-domain conformation even in the absence of an orthosteric agonist, whereas C4 behaves as a silent allosteric ligand that preserves the receptor’s ligand-dependent conformational transitions
7RNN	Human ASIC1a channel	Nanobody Nb.C1 (*Vicugna pacos*)	Nb.C1 binds the extracellular domain at a site overlapping with toxin-binding regions used by MitTx and mambalgin-1, without directly occluding the pore
7SSZ/8DFL/9CTY	Human Kv1.3 K^+^ channel	Nanobody A0194009G09 (*Lama glama*)	Rather than directly blocking the permeation pathway, A0194009G09 inhibits Kv1.3 by stabilizing a C-type-inactivated, nonconductive pore conformation, illustrating how extracellular nanobodies can control voltage-gated K^+^ channels through conformational trapping
8QZ1/8QZ2/8QZ3/8QZ4	TREK-2 (K2P10.1) K^+^ channel	Nanobodies Nb58/Nb61/Nb67/Nb76 (*Lama glama*)	This nanobody panel illustrates multiple modes of K2P-channel modulation: Nb58 acts primarily as a tight-binding structural binder, whereas Nb61, Nb67, and Nb76 are functionally active extracellular modulators, with Nb67 and Nb76 described as activator nanobodies
7P5V/7P5W 7P60/7P5Y/7P6K	Volume-Regulated Anion Channel LRRC8A	Sybodies Sb1/Sb2/Sb4/Sb3/Sb5 (*synthetic construct*)	Synthetic nanobody-like binders, termed sybodies, target the cytoplasmic leucine-rich repeat domain. These small synthetic scaffolds can allosterically inhibit or enhance LRRC8A activity by binding distinct LRR-domain epitopes and reshaping cytosolic-domain conformations coupled to channel gating
6HDC/6HD8/6HD9/6HDA/6HDB/6SWR	TMEM175 K^+^ channel	Nanobody-MBP fusion (*Lama glama*)	The nanobody–MBP fusion protein functions primarily as a crystallization chaperone by stabilizing a closed channel conformation, improving crystal diffraction, and enabling high-resolution analysis of the noncanonical TMEM175 ion-conduction pathway and K^+^-selectivity mechanism

Mapping this onto TRPM4 reveals an unusually rich set of targetable surfaces. Extracellularly, the EPGF-containing turret near the pore entrance is already validated as an inhibitory epitope (Wei et al., 2022) and is an obvious starting point for nanobody blockade. Intracellularly—accessible to nanobodies expressed as intrabodies (intracellular antibodies) or delivered by emerging intracellular-delivery strategies—the large MHR/NBD scaffold and the C-terminal umbrella present multifunctional regulatory hubs where Ca^2+^, PIP_2_, ATP, CaM, and S100A1 act on overlapping epitopes (Bousova et al., 2018; 2020; Vydra Bousova et al., 2023). A binder engineered to stabilize the ATP intersubunit “glue” interface, stabilize the PIP_2_-supported coupling geometry, or constrain the F910–F935 contact that enables productive coupling could, in principle, bias TRPM4 toward defined open or closed conformations—the molecular equivalent of selectively stabilizing specific states within the gating cycle. At present, however, this remains a structure-guided design hypothesis rather than a validated strategy for TRPM4; its feasibility will require experimental demonstration that such binders can engage the intended interfaces with high specificity, stabilize the predicted conformational states, and produce controlled modulation of channel activity.

In addition to their potential therapeutic use, nanobodies generated against specific conformational states would serve as invaluable markers and conformational locks for cryo-EM studies, where small, rigid binders are already used to enable high-resolution reconstruction of challenging membrane proteins (Bloch et al., 2021). It must be stated plainly that, for TRPM4 specifically, state- and site-selective nanobody modulation remains prospective rather than demonstrated (Ali et al., 2025); the precedents above, however, make it a credible and high-value goal rather than a speculative one.

Recent structural studies have captured several major functional states of TRPM4; this means that a nanobody or minibinder could be rationally designed to target, for example, the PIP_2_-coupling junction of the open state or the cytosolic ATP interface of the inhibited state, with the goal of selectively modulating channel function rather than relying solely on epitopes generated through conventional immunization approaches.

The convergence of state-resolved TRPM4 structural biology with binder design therefore points toward a new class of modulators: conformation- and site-selective nanobodies that act as molecular “state stabilizers,” stabilizing open conformations where activation is therapeutic—for instance, to trigger NECSO-type sodium-overload death in high-TRPM4 tumors—or stabilizing non-conductive states where depolarizing Na^+^ entry drives pathology, as in cerebral edema or selected arrhythmias, all with the selectivity that biologics afford. Despite these opportunities, important challenges remain. Intracellular epitopes require intrabody expression or advances in cytosolic delivery; species-specific epitopes complicate rodent validation, exactly as they do for existing antibodies; designed binders still demand rigorous experimental confirmation of affinity, specificity, and functional effect; and developability, immunogenicity, and pharmacokinetics must be established for any candidate intended for the clinic. None of these is unique to TRPM4, and each is the focus of intense methodological progress. The essential point is that TRPM4 may represent a promising test case for structure-targeted biologics—a channel whose disease relevance is established, for which several functionally relevant conformational states have been structurally characterized, and whose modulation requires the level of precision that nanobody engineering, structure-guided computational approaches, and emerging AI-based design strategies are increasingly able to provide.

### 5.2 Future directions for TRPM4-targeted therapies

The structural and pharmacological landscape of TRPM4 has reached a point where future work should move beyond the conventional distinction between channel blockers and activators. Resolved cryo-EM structures now show that TRPM4 does not operate through a single linear gating transition, but rather through a conformational ensemble shaped by Ca^2+^, PIP_2_, ATP, temperature, membrane lipids, and ligand occupancy. These conformations are best defined primarily by pore architecture, including closed, pre-open or non-conductive intermediate, and open states. Within this framework, apo TRPM4 adopts a closed conformation, Ca^2+^ binding can stabilize closed or pre-open intermediates, and pore opening can be promoted when Ca^2+^-dependent rearrangements are coupled to additional regulatory inputs such as PIP_2_ or the positive modulator decavanadate (DVT). Conversely, ATP and direct inhibitors can stabilize distinct non-conductive conformations, including closed or Ca^2+^-primed pre-open states, without defining “inhibited” as a separate structural category. Accordingly, the key therapeutic opportunity is not just to suppress or enhance TRPM4 activity, but to shift the channel towards specific functional states. These states are distinguished not only by pore diameter, but by the integrity of the allosteric pathway linking the S1–S4 sensor-like domain, the S4–S5 linker, the TRP helix, the S6 gate, and the cytosolic MHR/rib-helix scaffold.

This state-based framework has direct implications for drug discovery. Future TRPM4 modulators should be evaluated under conditions that preserve the regulatory context in which the channel actually functions: human TRPM4, physiological temperature, relevant Ca^2+^ and nucleotide concentrations, PIP_2_-containing membranes, and native-like lipid environments whenever possible. Screening campaigns performed at low temperature, in lipid-depleted systems, or using non-human orthologs may misclassify ligand activity, overlook state-dependent agonists or inhibitors, and underestimate species-specific pharmacology. For this reason, electrophysiology, structural biology, mutagenesis, molecular dynamics, ligand-binding assays, trafficking measurements, and disease-relevant cellular models should be integrated early rather than treated as sequential validation steps.

A second priority is to establish rigorous pharmacological benchmarks. TRPM4 research has benefited from classical tool compounds such as flufenamic acid, 9-phenanthrol, and glibenclamide-sensitive SUR1–TRPM4 paradigms, but these approaches often suffer from limited selectivity, indirect mechanisms, or context-dependent interpretation. Newer anthranilic-acid inhibitors, direct agonists such as NC1 and DAB, ligands whose binding affinity is modulated by the temperature-dependent conformational state of TRPM4, and extracellular antibodies provide a more mechanistically informative foundation. However, each candidate should be classified according to binding site, state preference, ortholog sensitivity, acute versus chronic effects, and functional consequence in native cells. Such benchmarking will be essential to distinguish true TRPM4 modulation from altered expression, trafficking, partner-protein regulation, or compensatory changes in Ca^2+^ signaling.

Therapeutic prioritization should remain disease-context dependent. Acute CNS injury and edema currently provide the clearest near-term rationale because TRPM4 or SUR1–TRPM4 activity is induced within a defined pathological window and is linked to ionic imbalance, depolarization, osmotic stress, and secondary tissue injury. Cardiovascular disease requires a more cautious strategy because both gains and losses of TRPM4 function can disturb excitability and conduction (Amarouch and El Hilaly, 2020); intervention in this setting will likely require genotype-aware and tissue-specific approaches. Cancer and inflammatory diseases are attractive but less mature therapeutic areas. In these contexts, the key unresolved issue is whether TRPM4 acts as a causal driver, a biomarker of pathological remodeling, or a conditional vulnerability that becomes therapeutically relevant only in defined cellular, metabolic, or microenvironmental states. Addressing this question will require stronger disease models, cleaner pharmacological tools, and direct tests of how TRPM4-dependent depolarization intersects with proliferation, survival, migration, cytokine production, immune-cell activation, and cell-death pathways.

Beyond small molecules, biologics and engineered binders offer an important route toward selectivity. Extracellular antibodies have already shown that TRPM4 presents accessible inhibitory surfaces, and nanobodies or AI-designed minibinders could further expand this concept by recognizing extracellular epitopes, conformationally selective surfaces, or disease-associated channel assemblies. These modalities may be particularly useful for stabilizing non-conductive states, trapping open-competent intermediates, or selectively targeting pathological TRPM4 populations at the cell surface. Nevertheless, binder-based modulation of TRPM4 remains an emerging strategy. Each candidate must be validated for affinity, epitope accessibility, ortholog specificity, conformational bias, effects on gating versus trafficking, delivery feasibility, immunogenicity risk, and functional efficacy under physiological membrane conditions. Computational and AI-assisted protein-design approaches may accelerate discovery and optimization, but they should be viewed, at present, as enabling technologies rather than as established TRPM4-specific therapeutic platforms.

Overall, the next phase of TRPM4 research should be defined by convergence. Structural biology can identify conformational states and druggable surfaces; computational biology can model state transitions, lipid-dependent allostery, and ligand-induced energy landscapes; electrophysiology can determine whether these states correspond to meaningful changes in channel function; and disease biology can define when TRPM4 activity is harmful, adaptive, or merely correlative. The field is now positioned to ask a more precise therapeutic question: not whether TRPM4 can be modulated, but which TRPM4 state should be stabilized, in which disease context, and by which molecular modality.

## 6 Discussion

TRPM4 has evolved from a broadly studied Ca^2+^-activated conductance into a structurally interpretable and therapeutically relevant ion channel. Recent structural advances have transformed the mechanistic understanding of this interface. TRPM4 is now understood as a multi-state allosteric channel. TRPM4 has emerged as a structurally defined, disease-relevant ion channel that converts intracellular Ca^2+^ signals into membrane depolarization through monovalent cation flux. This function places TRPM4 at a key intersection among Ca^2+^ signaling, excitability, osmotic balance, immune responses, and cell death pathways. Recent structural and pharmacological studies indicate that TRPM4 should not be seen merely as a channel to block or activate. Instead, it is a multi-state allosteric system controlled by Ca^2+^, PIP_2_, ATP, temperature, lipids, and ligand binding. Therefore, the main therapeutic challenge is to stabilize the appropriate TRPM4 state in the appropriate disease context. Future progress will depend on state-selective pharmacology, human-relevant models, careful validation across orthologs, and integration of structural biology, electrophysiology, computational modeling, and disease studies.

Within this framework, the next-generation of TRPM4 therapeutics should move beyond simple channel blockade toward state-selective modulation. Small molecules, extracellular antibodies, nanobodies, and computationally designed binders offer complementary routes for engaging defined regulatory surfaces and stabilizing therapeutically useful conformations. Thus, TRPM4 stands as a compelling test case for mechanism-guided TRP-channel pharmacology and a potential therapeutic target in disorders driven by maladaptive depolarizing Na^+^ influx, provided that future modulators combine molecular specificity with context-dependent efficacy.
